# Infections in early life and childhood leukaemia risk: a UK case–control study of general practitioner records

**DOI:** 10.1038/sj.bjc.6604696

**Published:** 2008-09-30

**Authors:** C R Cardwell, P A McKinney, C C Patterson, L J Murray

**Affiliations:** 1 Department of Epidemiology and Public Health, School of Medicine and Dentistry, The Queen's University of Belfast, Belfast, UK; 2 Cancer Epidemiology and Prevention Research Group, The Queen's University of Belfast, Belfast, UK; 3 Paediatric Epidemiology Group, Centre for Epidemiology and Biostatistics, University of Leeds, UK

**Keywords:** epidemiology, infection, leukaemia, acute, childhood

## Abstract

We investigated infections in early life (diagnosed in general practice) and subsequent risk of childhood leukaemia in the UK General Practice Research Database (GPRD). All children born at GPRD practices and subsequently diagnosed with leukaemia were identified as cases and were individually matched (on year of birth, sex and practice) to up to 20 controls. The final analysis included 162 leukaemia cases and 2215 matched controls. Conditional logistic regression demonstrated no evidence that children with one or more recorded infection in the first year of life had a reduced risk of leukaemia (OR=1.05, 95%CI 0.69, 1.59; *P*=0.83) or acute lymphoblastic leukaemia (ALL; OR=1.05, 95%CI 0.64–1.74; *P*=0.84). Our study provides no support for the Greaves hypothesis, which proposes that reduced or delayed exposure to infections in early life increases the risk of childhood ALL.

The Greaves’ hypothesis (Greaves, 2006) suggests that reduced or delayed exposure to infection at an early age increases the risk of an abnormal immune response potentially precipitating the onset of childhood acute lymphoblastic leukaemia (ALL). In support of this theory, various studies have demonstrated associations with various proxy measures of exposure to infection, such as day care attendance (Perrillat *et al*, 2002; Jourdan-Da Silva *et al*, 2004; Ma *et al*, 2005) and breastfeeding (UK Childhood Cancer Study Investigators, 2001). However, only one study has previously reported on ALL in relation to routinely recorded infections in early life (Roman *et al*, 2007). Other investigations of leukaemia and infections in early life (Dockerty *et al*, 1999; Chan *et al*, 2002; Perrillat *et al*, 2002; Jourdan-Da Silva *et al*, 2004; Ma *et al*, 2005; Macarthur *et al*, 2008) have primarily ascertained infection from parental recall, but this has been reported to be an inaccurate method for collecting data on childhood infections (McKinney *et al*, 1991). Furthermore, the parental recall of infections is open to disease-dependent bias as demonstrated by a recent study, which observed that the degree of underreporting of infections that were recorded in GP records differed in parents of children with leukaemia compared to parents of healthy children (Simpson *et al*, 2007).

We used the General Practice Research Database (GPRD) to investigate any association between clinically diagnosed infections (and prescribed antibiotics) in the first year of life and childhood leukaemia and its main subtype ALL.

## Materials and methods

The GPRD, established in 1987, is one of the world's largest databases of anonymised longitudinal medical records from primary care, holding information on over 3 million patients (from over 400 practices) across the United Kingdom. Participating practices are audited to ensure that patient records include 95% of prescribing and relevant patient encounter events. Prescriptions are coded on the database with Prescription Pricing Authority codes. Medical conditions are coded with a modification of the Oxford Medical Information System (OXMIS) classification and READ codes (Kinn *et al*, 1995). The diagnostic and prescription data recorded in the GPRD has been validated by several studies (Walley and Mantgani, 1997; Hansell *et al*, 1999). Ethical approval for all observational research using GPRD data has been obtained from a Multicentre Research Ethics Committee (MREC).

A cohort was established containing children born at GPRD practices since the inception of the GPRD in 1987, excluding those without a recorded birth month, for whom the first year of life (the exposure period of interest) could not be determined. Cases were identified from the cohort if their records contained any diagnosis for leukaemia. Controls were also selected from this cohort and matched to individual cases by practice, gender and year of birth. For each case, up to 20 controls were randomly selected from the available matches in the cohort. Data were extracted from the GPRD in August 2007.

Cases and controls were excluded if they were not registered on the GPRD within 3 months of birth, as registration rates after 3 months were low and stable consistent with routine reregistration because of changing GPs. This 3-month cutoff was applied for the same reasons in two previous GPRD studies of infections in early life (McKeever *et al*, 2002; Cardwell *et al*, 2008). Additionally, cases and controls were excluded if they had a diagnosis for Down’s syndrome as it is associated with childhood infection rates and leukaemia (Roman *et al*, 2007).

A separate analysis was conducted on a subgroup of cases classified as ALL, including diagnoses of acute lymphoid (lymphatic) leukaemia and lymphoid (lymphatic) leukaemia. Lists of OXMIS and READ codes for leukaemia and ALL were agreed by the authors.

Comprehensive lists of OXMIS and READ codes were assembled for infection and symptoms usually associated with infection (which included diarrhoea, fever, pyrexia, sore throat, earache, snuffles, vomiting and diarrhoea, dysuria, otorrhoea and chesty cough). These lists were manually checked by the authors and are available from the authors on request. A case or control was considered to have an infection (in the first year of life), if they had a consultation at which an infection code was recorded. Over 90% of consultations in the first year of life in which an infection code was recorded involved direct contact with a GP (such as clinic visits and house calls) or other health professional (such as hospital discharge details and letters from outpatients and so on), but other consultations were also considered relevant (such as telephone calls). A count of the number of consultations for infection was extracted for the first year of life. In cases diagnosed in the first year of life infections were only counted before leukaemia diagnosis. Similarly, in the matched controls, infections were only counted before the date of the leukaemia diagnosis of the reference case. Multiple consultations for an infection on the same day were only counted once to reduce the repeated counting of the same infection. For exploratory analyses, where possible, infection codes were categorised into the following groups: upper respiratory tract, lower respiratory tract, otitis media, conjunctivitis, gastrointestinal, urinary tract and non-invasive fungal disease. A separate analysis was also conducted on chicken pox as a prior hypothesis existed for this exposure (Adelstein and Donovan, 1972; Bithell *et al*, 1973; Vianna and Polan, 1976).

A list of antibiotic codes was assembled from the GPRD medicine codes, which comprised section 5.1 of the *British National Formulary (BNF*; British Medical Association and the Royal Pharmaceutical Society of Great Britain, 2006). A wider list of codes for anti-infective medicines was also assembled comprising the entirety of chapter 5 of the *BNF*, excluding section 5.4.1 ‘Antimalarials’. A separate list of diagnosis codes was also assembled for chloramphenicol (ear and eye drops) as an earlier study had shown an association with chloramphenicol antibiotics (Shu *et al*, 1987). A count of the number of occurrences of these codes in the first year of life was then extracted from GPRD prescription data as described for infections.

### Statistical analysis

Initially, the proportions of cases and controls with recorded infections and prescribed antibiotics in the first year of life were calculated. Conditional logistic regression was then used to calculate odds ratios (ORs), and 95% confidence intervals (95%CIs), to compare cases and controls with respect to infections and antibiotics. Various sensitivity analyses were conducted. To adjust for the propensity to consult a GP, a variable counting non-infection-related consultations in the first year of life was added to the conditional logistic regression model. An analysis was also conducted restricting cases and controls to those registered on the GPRD within a month of birth, rather than 3 months, to investigate any impact of the delay between the birth and registration. A final sensitivity analysis was conducted, including only cases diagnosed after their second birthday to ensure that the delay between infection in the first year of life and leukaemia diagnosis was at least 12 months.

A power calculation was conducted before analysis. In an unmatched analysis comparing 160 cases of childhood leukaemia with 14 matched controls per case assuming 75% of controls had at least one recorded infection in the first year of life, the study would have approximately 80% power to detect as significant at the 5% level a reduction in the risk of leukaemia of 40% (an OR of 0.60) in children with at least one recorded infection in the first year of life.

All statistical analyses were performed using STATA release 8.0 (Stata Corporation, College Station, TX, USA).

## Results

Originally, 245 cases and 4381 age, sex and practice-matched controls were identified in the cohort of children born between 1987 and 2005 at GPRD practices for whom birth month was recorded. Children with Down’s syndrome were then excluded leaving 236 cases and 4210 controls. After the exclusion of participants registered after 3 months of birth, the final data set included 162 cases of childhood leukaemia, 112 of whom had ALL, and 2125 matched controls. The numbers of matched controls for each case ranged from 1 to 20 (mean=14, s.d.=5).

Table 1 contains patient characteristics of the cases, ALL cases and matched controls used in the analysis. The majority of cases of leukaemia occurred in the first 5 years of life. A comparable percentage of cases and controls had at least one consultation with their GP in the first year of life (94 and 95%, respectively). The mean number of consultations in the first year was slightly greater in cases than in controls (8.8 *vs* 8.3). Correspondingly, the mean number of direct consultations (consisting only of GP surgery, clinic visits and house calls) was also slightly greater in cases than controls (8.0 *vs* 7.5). The ALL cases displayed a similar pattern.

A comparison of consultations for infections in the first year of life between cases, ALL cases and controls is summarised in Table 2. The percentage with at least one consultation for an infection in the first year of life was similar in cases and controls (63 and 62%, respectively) as with ALL (64 and 65%, respectively). There was some evidence of a difference in the distribution of the number of consultations for infection between cases and controls (*P*=0.03) and between the ALL cases and their controls (*P*=0.01). Specifically, there were indications that children with three or more infections in the first year of life had a slightly increased risk of leukaemia (and ALL) compared with other children, but peculiarly there was a slight, though not significant, reduction in the risk of leukaemia (and ALL) in children with one or two infections in the first year of life compared to children with none. The analysis of infections and associated symptoms are shown in Supplementary Table 1 (available online) and produced broadly similar conclusions.

Table 2 also shows the association between various common childhood infections in the first year of life (including upper respiratory tract, lower respiratory tract, otitis media, conjunctivitis, gastrointestinal, urinary tract and fungal) and leukaemia and ALL risk. There was some evidence of an increased risk of leukaemia following upper respiratory tract infections (OR=1.56, *P*=0.02) and chicken pox (OR=2.41, *P*=0.02). Slightly greater increases in ALL risk were observed after upper respiratory tract infections (OR=1.59, *P*=0.04) and chicken pox (OR=2.62, *P*=0.04). There was little evidence of associations between leukaemia or ALL risk and any of the other recorded infections in the first year of life.

### Antibiotics

Details of prescriptions for antibiotics in the first year of life in cases and controls are shown in Table 3, with some suggestions, though not significant, of a greater proportion in cases than controls (52 *vs* 47%, respectively, *P*=0.11) as with ALL (54 *vs* 50% respectively, *P*=0.17). There was no association between the number of prescriptions for antibiotics and leukaemia risk (*P* for trend=0.47) or ALL risk (*P* for trend=0.34). A similar pattern was observed when all anti-infectives in the first year of life were compared between the cases, ALL cases and controls, and is shown in Supplementary Table 1. Finally, there was no association between prescriptions for chloramphenicol ear or eye drops in the first year of life and leukaemia or ALL risk.

### Sensitivity analyses

In all sensitivity analyses (after adjustment for non-infection-related consultations, including only cases and controls registered at their GP within 1 month of birth and including only cases diagnosed with leukaemia after their second birth day), the main findings for infections and antibiotics were broadly similar (data not shown).

## Discussion

This study provides no evidence of an increased risk of leukaemia or ALL in children with fewer clinically recorded infections or prescriptions for antibiotics in the first year of life. Consequently, our results do not support the Greaves hypothesis (Greaves, 2006).

The main strength of this study is that consultations for infections with dates and prescribed antibiotics in early life were routinely recorded by the GP before the onset of disease, reducing the possibility of recall or selection biases.

This study has a number of weaknesses. First, the GPRD database of primary care records is likely to underestimate the number of infections experienced by a child in the first year of life as parents may not consult the GP for every infection, particularly less severe infections. This is unlikely to influence our findings appreciably as there seems no obvious reason for underreporting to differ between cases and controls. Another weakness was the delay between the dates of birth and GPRD registration of cases and controls, which could have lead to the under-ascertainment of infections in the first few months of life. However, in the main analysis, this delay was identical between cases and controls, and the findings were little altered when the analysis was restricted to children registered within a month of birth. Also, as with all observational studies, it is not possible to rule out the effect of confounding by unrecorded variables such as day care attendance and breastfeeding.

Our findings are in agreement with the only other case control study of leukaemia in relation to routinely recorded infections in the first year (Roman *et al*, 2007); both found no evidence of a reduced risk of leukaemia or ALL. Other recent studies have ascertained infections primarily from parental recall. The majority of these studies have observed slight inverse associations between infections or specific types of infections in early life and leukaemia risk (Neglia *et al*, 2000; Perrillat *et al*, 2002; Jourdan-Da Silva *et al*, 2004; Ma *et al*, 2005), but some studies have demonstrated little evidence of an association (Schuz *et al*, 1999; Macarthur *et al*, 2008), whereas others have shown an increased risk of leukaemia after infection (Dockerty *et al*, 1999; Chan *et al*, 2002). However, parental recall of infections is open to disease-dependent recall bias (Simpson *et al*, 2007) and has been shown to be an unreliable measure of both the timing and occurrence of infections (McKinney *et al*, 1991).

The findings for specific infections should be interpreted cautiously as eight infections were investigated increasing the risk of detecting spurious associations by chance. However, it is interesting that an increased leukaemia and ALL risk after a URTI infection in the first year of were also recorded by a recent UK study (Roman *et al*, 2007). With respect to an earlier hypothesis (Adelstein and Donovan, 1972; Bithell *et al*, 1973; Vianna and Polan, 1976), we found a significant increase in the risk of developing leukaemia and ALL after chicken pox. Although on the basis of relatively few cases, this association is unbiased by parental recall, and therefore warrants further investigation. If these observed increases in the risk of leukaemia are real, it is unclear whether these infections are causative (Roman *et al*, 2007) or whether subtle immunodeficiency underlies increases both in early life infections and ALL risk (Dorak *et al*, 2007).

In this study, no participant was prescribed oral chloramphenicol, previously reported as associated with leukaemia risk (Shu *et al*, 1987). However, many children were prescribed chloramphenicol eyeear drops in the first year of life and these were not associated with leukaemia risk.

In conclusion, this study provides little evidence of a reduction in the risk of ALL or leukaemia, after clinically diagnosed infections in the first year of life, and therefore is not consistent with the Greaves hypothesis.

## Figures and Tables

**Table 1:** Characteristics of cases with childhood leukaemia (*n*=162) and matched controls (*n*=2125), all registered within 3 months of birth

	**Any leukaemia**		**ALL**		**Controls**	
**Characteristics**	**Mean (s.d.)**	**No*.* (%)**	**Mean (s.d.)**	**No*.* (%)**	**Mean (s.d.)**	**No.** **(%)**
	*(n=162)*		*(n=112)*		*(n=2125)*	
*Sex*						
Male		83 (51)		58 (52)		1086 (51)
Female		79 (49)		54 (48)		1039 (49)
						
*Age at diagnosis*						
0–4		109 (67)		75 (67)		
5–9		38 (23)		26 (23)		
10–14		15 (9)		11 (10)		
						
*GP consultations in the first year of life*						
At least one consultation^a^		152 (94)		105 (94)		2023 (95)
*Number of consultations* a	8.8 (7.3)		9.1 (7.5)		8.3 (7.0)	
0–4		54 (33)		33 (29)		725 (34)
5–9		48 (30)		36 (32)		703 (33)
10–14		26 (16)		19 (17)		388 (18)
15–19		17 (10)		13 (12)		163 (8)
⩾20		17 (10)		11 (10)		146 (7)
						
At least one direct consultation^b^		152 (94)		105 (94)		2016 (95)
						
*Number of direct consultations* b	8.0 (6.3)		8.2 (6.4)		7.5 (6.1)	
0–4		63 (39)		41 (37)		800 (38)
5–9		43 (27)		30 (27)		722 (34)
10–14		28 (17)		20 (18)		366 (17)
15–19		17 (10)		14 (13)		135 (6)
⩾20		11 (7)		7 (6)		102 (5)

a Includes GP surgery consultations, clinic consultations, house calls, phone calls, mail and other administration, and excludes consultations for immunisations.

b Includes GP surgery consultations, clinic consultations and house calls, and excludes consultations for immunisations.

**Table 2:** Consultations for infection in the first year of life in children with leukaemia and matched controls

	**Any childhood leukaemia**				**Childhood acute lymphoblastic leukaemia**			
	**Cases**	**Controls**			**Cases**	**Controls**		
**Consultations**	**No. (%)**	**No. (%)**	**OR (95%CI)**	***P*-value**	**No. (%)**	**No. (%)**	**OR (95%CI)**	***P*-value**
	*(n=162)*	*(n=2125)*			*(n=112)*	*(n=1435)*		
At least one consultation for infection	102 (63)	1323 (62)	1.05 (0.69, 1.59)	0.83	72 (64)	928 (65)	1.05 (0.64, 1.74)	0.84
*Total consultations for infections*								
0	60 (37)	802 (38)	1.00	0.03^a^	40 (36)	507 (35)	1.00	0.01^a^
1–2	46 (28)	764 (36)	0.84 (0.53, 1.34)	(0.08)^b^	29 (26)	544 (38)	0.78 (0.44, 1.37)	(0.05)^b^
⩾3	56 (35)	559 (26)	1.48 (0.91, 2.43)		43 (38)	384 (27)	1.70 (0.94, 3.08)	
Mean (s.d.)	2.0 (2.4)	1.8 (2.3)	1.06 (0.98, 1.13)^c^	0.14^c^	2.3 (2.6)	1.9 (2.4)	1.08 (1.00, 1.16)^c^	0.08^c^
								
*At least one consultation for*								
Upper respiratory tract infections	79 (49)	859 (40)	1.56 (1.08, 2.27)	0.02	56 (50)	602 (42)	1.59 (1.02, 2.49)	0.04
Lower respiratory tract infections	11 (7)	129 (6)	1.22 (0.62, 2.39)	0.56	9 (8)	94 (7)	1.42 (0.67, 3.04)	0.36
Otitis media	14 (9)	231 (11)	0.74 (0.41, 1.34)	0.31	11 (10)	167 (12)	0.83 (0.42, 1.64)	0.59
Conjunctivitis	35 (22)	449 (21)	1.06 (0.70, 1.60)	0.79	30 (27)	317 (22)	1.39 (0.87, 2.22)	0.17
Gastrointestinal infections	15 (9)	125 (6)	1.69 (0.93, 3.06)	0.08	11 (10)	79 (6)	1.89 (0.93, 3.84)	0.10
Urinary tract infections	1 (1)	21 (1)	0.53 (0.07, 4.08)	0.54	1 (1)	17 (1)	0.64 (0.08, 5.10)	0.66
Fungal infections	16 (10)	214 (10)	0.95 (0.54, 1.66)	0.85	11 (10)	153 (10)	0.85 (0.43, 1.66)	0.62
Chicken pox	9 (6)	50 (2)	2.41 (1.14, 5.09)	0.02	7 (6)	37 (3)	2.62 (1.12, 6.13)	0.04

a Test for difference in odds between categories.

b Test for trend across categories.

c Odds of disease per infection increase and associated *P*-value.

**Table 3:** Prescriptions for antibiotics in the first year of life in children with leukaemia and matched controls

	**Any childhood leukaemia**				**Childhood acute lymphoblastic leukaemia**			
	**Cases**	**Controls**			**Cases**	**Controls**		
**Prescription**	**No. (%)**	**No. (%)**	**OR (95%CI)**	***P*-value**	**No. (%)**	**No. (%)**	**OR (95%CI)**	***P*-value**
	*(n=162)*	*(n=2125)*			*(n=112)*	*(n=1435)*		
At least one prescription for an antibiotic	84 (52)	1000 (47)	1.34 (0.93, 1.94)	0.11	60 (54)	713 (50)	1.36 (0.87, 2.13)	0.17
								
*Antibiotic prescribing frequency*								
0	78 (48)	1125 (53)	1.00	0.11^a^	52 (46)	722 (50)	1.00	0.35^a^
1–2	68 (42)	737 (35)	1.45 (0.99, 2.10)	0.47^b^	45 (40)	509 (35)	1.41 (0.88, 2.24)	0.34^b^
⩾3	16 (10)	263 (12)	0.98 (0.53, 1.78)		15 (13)	204 (14)	1.22 (0.63, 2.37)	
Mean (s.d.)	1.0 (1.6)	1.0 (1.5)	1.05 (0.95, 1.15)^c^	0.48^c^	1.2 (1.8)	1.1 (1.6)	1.06 (0.95, 1.19)^c^	0.33^c^
At least one prescription for chloramphenicol (eareye drops)	29 (18)	408 (19)	0.92 (0.59, 1.42)	0.72	22 (20)	264 (18)	1.14 (0.68, 1.90)	0.63

a Test for difference in odds between categories.

b Test for trend across categories.

c Odds of disease per prescription increase and associated *P*-value.
