# Supplementary material for: Infections in early life and childhood leukaemia risk: a UK case–control study of general practitioner records
Source: Br J Cancer. 2008 Sep 30;99(9):1529–33. doi: 10.1038/sj.bjc.6604696 (PMC2579698; doi:10.1038/sj.bjc.6604696)
Supplement: Supplementary Table 1 [file 6604696x1.doc]

Supplementary Table 1.

| **Consultations \ Prescriptions** | | **Any childhood leukaemia** | | | |  | | **Childhood acute lymphoblastic leukaemia** | | | |
| --- | --- | --- | --- | --- | --- | --- | --- | --- | --- | --- | --- |
|  |  | Cases | Controls | OR (95%CI) | P | | Cases | | Controls | OR (95%CI) | P |
|  |  | Nos. (%) | Nos. (%) |  |  |  | Nos. (%) | | Nos. (%) |  |  |
|  | | *(n=162)* | *(n=2,125)* |  |  | | *(n=112)* | | *(n=1,435)* |  |  |
|  | |  |  |  |  | |  | |  |  |  |
| At least one consultation for infection or associated symptoms of infection | | 110 (68) | 1410 (66) | 1.12 (0.71, 1.76) | 0.63 | | 76 (68) | | 993 (69) | 0.98 (0.57, 1.67) | 0.93 |
|  | |  |  |  |  | |  | |  |  |  |
| Total consultations for infections  or symptoms associated with infection | | |  |  |  | |  | |  |  |  |
|  | 0 | 52 (32) | 715 (34) | 1.00 | 0.25^*^ | | 36 (32) | | 442 (31) | 1.00 | 0.16^*^ |
|  | 1-2 | 47 (29) | 702 (33) | 0.98 (0.60, 1.60) | (0.17^†^) | | 29 (26) | | 497 (35) | 0.80 (0.44, 1.45) | (0.26^†^) |
|  | ≥ 3 | 63 (39) | 708 (33) | 1.37 (0.82, 2.30) |  | | 47 (42) | | 496 (35) | 1.30 (0.70, 2.42) |  |
|  |  |  |  |  |  | |  | |  |  |  |
|  | Mean (sd) | 2.5 (2.9) | 2.2 (2.7) | 1.06 (1.00, 1.13)^‡^ | 0.08^‡^ | | 2.8 (3.1) | | 2.3 (2.8) | 1.08 (1.00, 1.15)^‡^ | 0.05^‡^ |
|  | |  |  |  |  | |  | |  |  |  |
|  | |  |  |  |  | |  | |  |  |  |
| At least one prescription for an anti-infective | | 88 (54) | 1073 (50) | 1.27 (0.88, 1.82) | 0.19 | | 62 (55) | | 763 (53) | 1.22 (0.79, 1.89) | 0.37 |
|  | |  |  |  |  | |  | |  |  |  |
| Anti-infective prescribing frequency | | |  |  |  | |  | |  |  |  |
|  | 0 | 74 (46) | 1052 (50) | 1.00 | 0.31^*^ | | 50 (45) | | 672 (47) | 1.00 | 0.79^*^ |
|  | 1-2 | 67 (41) | 771 (36) | 1.33 (0.91, 1.93) | (0.46^†^) | | 44 (39) | | 536 (37) | 1.22 (0.77, 1.93) | (0.44^†^) |
|  | ≥ 3 | 21 (13) | 302 (14) | 1.08 (0.62, 1.87) |  | | 18 (16) | | 227 (16) | 1.22 (0.66, 2.27) |  |
|  |  |  |  |  |  | |  | |  |  |  |
|  | Mean (sd) | 1.2 (1.7) | 1.1 (1.6) | 1.03 (0.91, 1.18)^‡^ | 0.62^‡^ | | 1.3 (1.9) | | 1.2 (1.8) | 1.06 (0.96, 1.18) ^‡^ | 0.25^‡^ |
|  | |  |  |  |  | |  | |  |  |  |

*Test for difference in odds between categories.  ^†^ Test for trend across categories. ^‡^Odds of disease per prescription increase and associated P-value.
